# Recurrent internal tandem duplications of *BCOR* in clear cell sarcoma of the kidney

**DOI:** 10.1038/ncomms9891

**Published:** 2015-11-17

**Authors:** Angshumoy Roy, Vijetha Kumar, Barry Zorman, Erica Fang, Katherine M. Haines, HarshaVardhan Doddapaneni, Oliver A. Hampton, Simon White, Abhishek A. Bavle, Nimesh R. Patel, Karen W. Eldin, M. John Hicks, Dinesh Rakheja, Patrick J. Leavey, Stephen X. Skapek, James F. Amatruda, Jed G. Nuchtern, Murali M. Chintagumpala, David A. Wheeler, Sharon E. Plon, Pavel Sumazin, D. Williams Parsons

**Affiliations:** 1 Department of Pathology & Immunology, Baylor College of Medicine, Houston, Texas 77030, USA; 2 Department of Pathology, Texas Children's Hospital, Houston, Texas 77030, USA; 3 Texas Children's Cancer Center, Houston, Texas 77030, USA; 4 Department of Pediatrics, Baylor College of Medicine, Houston, Texas 77030, USA; 5 Dan L. Duncan Cancer Center, Baylor College of Medicine, Houston, Texas 77030, USA; 6 Department of Molecular and Human Genetics, Baylor College of Medicine, Houston, Texas 77030, USA; 7 Human Genome Sequencing Center, Baylor College of Medicine, Houston, Texas 77030, USA; 8 Department of Pathology, University of Texas Southwestern Medical Center, Dallas, Texas 75390, USA; 9 Department of Pathology and Laboratory Medicine, Children's Medical Center, Dallas, Texas 75390, USA; 10 Department of Pediatrics, University of Texas Southwestern Medical Center, Dallas, Texas 75390, USA; 11 Division of Pediatric Surgery, Michael E. DeBakey Department of Surgery, Baylor College of Medicine, Houston, Texas 77030, USA; 12 Division of Pediatric Surgery, Department of Surgery, Texas Children's Hospital, Houston, Texas 77030, USA

## Abstract

The X-linked BCL-6 co-repressor (*BCOR*) gene encodes a key constituent of a variant polycomb repressive complex (PRC) that is mutated or translocated in human cancers. Here we report on the identification of somatic internal tandem duplications (ITDs) clustering in the C terminus of *BCOR* in 23 of 27 (85%) pediatric clear cell sarcomas of the kidney (CCSK) from two independent cohorts. We profile CCSK tumours using a combination of whole-exome, transcriptome and targeted sequencing. Identical ITD mutations are found in primary and relapsed tumour pairs but not in adjacent normal kidney or blood. Mutant *BCOR* transcripts and proteins are markedly upregulated in ITD-positive tumours. Transcriptome analysis of ITD-positive CCSKs reveals enrichment for PRC2-regulated genes and similarity to undifferentiated sarcomas harbouring *BCOR*–*CCNB3* fusions. The discovery of recurrent *BCOR* ITDs defines a major oncogenic event in this childhood sarcoma with significant implications for diagnostic and therapeutic approaches to this tumour.

## Abstract (web summary)

The genetic basis of clear cell sarcomas of the kidney is not well understood. In this study, Roy *et al*. perform whole-exome and RNA sequencing of these tumours and identify recurrent internal tandem duplications in BCOR, a key constituent of a variant polycomb repressive complex.

## Graphical abstract

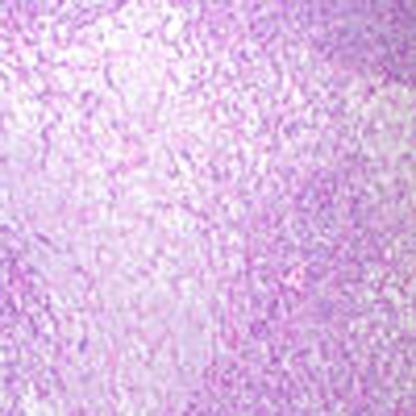

CCSK is a high-risk childhood cancer that comprises 2–5% of primary renal tumours diagnosed in children^1^,^2^. Although first distinguished as a clinicopathologic entity from the more common Wilms tumour in the 1970s (ref. 3), it remains a biologically and clinically ill-defined neoplasm^2^,^4^. Children with CCSK are predominantly young and male (median age 3 years; male to female ratio of >2:1), and the tumour is notable for late recurrences and metastases to bone and brain^1^,^2^,^5^. Although current intensive treatment regimens have resulted in improved outcomes for children with CCSK, survival for patients with relapsed tumours remains poor^1^,^2^.

Histologically, CCSKs are characterized by a diversity of morphological patterns that can confound accurate diagnosis in up to a quarter of cases^2^,^5^. Genetic studies to date have been generally unrevealing, with a *t*(10;17)(q22;p13) translocation resulting in *YWHAE*–*NUTM2* gene family fusions in a minority (12%) of cases as the only recurrent somatic aberration reported^6^. Unlike Wilms tumour, CCSK is not associated with familial cancer predisposition syndromes, suggesting that the genetic drivers for these tumours remain to be discovered^4^,^5^.

Here using whole-transcriptome sequencing (RNA-seq) and whole-exome sequencing (WES), we report on the identification of highly recurrent internal tandem duplications (ITDs) in the X-linked BCL-6 co-repressor (*BCOR*) gene that is mutated in various human cancers^7^,^8^,^9^,^10^. These genomic data provide insight into the biological processes perturbed in *BCOR*-mutated CCSKs and reveal unexpected similarities between CCSKs and soft-tissue sarcomas harbouring *BCOR*–*CCNB3* fusions.

## Results

### Identification of recurrent somatic ITDs in *BCOR*

To identify recurrent genomic aberrations in CCSK, we performed whole-transcriptome paired-end sequencing (RNA-seq) of three fresh-frozen tumour samples (cases 347T, 383T and 385T) and WES of one of the tumours and its matched peripheral blood sample as part of a clinical genomics study^11^ (Supplementary Table 1 and Supplementary Data 1). No recurrent in-frame fusions, fusions targeting known cancer genes or *YWHAE*–*NUTM2* fusions were identified by RNA-seq (Supplementary Data 2). However, WES of tumour 347T identified a putative stop-loss variant within the terminal coding exon 15 of the *BCOR* gene on Xp11.4 that was absent in the matched germline reads (Supplementary Fig. 1a and Supplementary Table 2).

**Figure 1:**
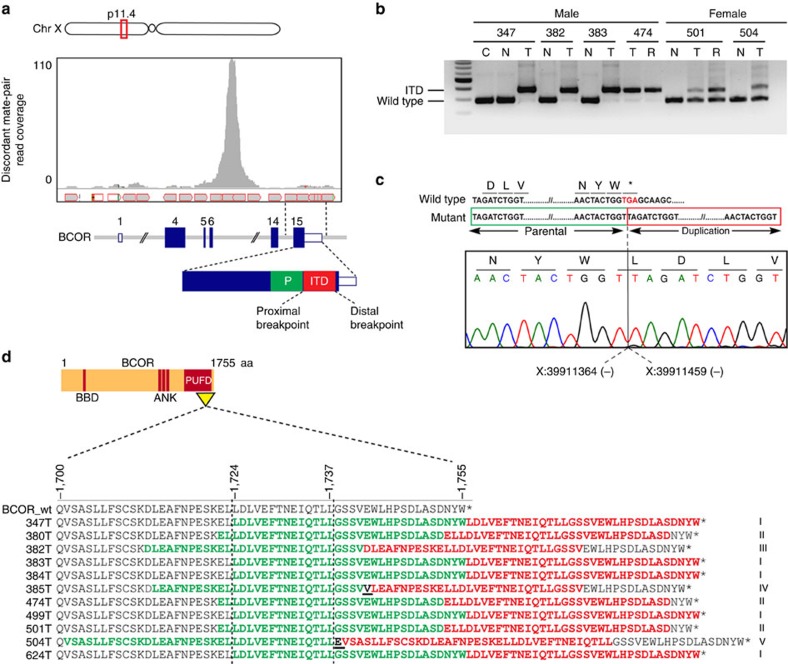
Recurrent somatic ITDs in the *BCOR* gene in CCSKs. (**a**) View of aligned whole-transcriptome sequencing reads from a single ITD-positive CCSK (347T) demonstrating a marked focal increase in read coverage corresponding to the ITD in exon 15 of *BCOR* on Xp11.4. Only unpaired reads of discordant mate pairs were used for local realignment. A representation of the ITD within the *BCOR* gene is shown beneath. The parental segment (P) that is duplicated is depicted in green and the tandem duplicated segment (ITD) is shown in red. (**b**) Targeted PCR and gel electrophoresis of *BCOR* exon 15 in samples from four representative male and two female subjects showed the expected wild-type products (288 bp) in the peripheral blood (C) and adjacent normal kidney (N) tissues, and larger products corresponding to the ITDs (87–114 bp) in the primary CCSK tumours (T) and relapsed metastatic tumours (R). Nearly undetectable levels of the wild-type products were observed in tumour samples from males; in females, both ITD-positive and wild-type products were evident. (**c**) Sanger sequence trace from case 347T showing the immediate sequence context surrounding the proximal genomic breakpoint in *BCOR* exon 15 (hg19 coordinates, negative strand). The wild-type genomic sequence around the breakpoint including the termination codon and 3′-UTR are shown above and the parental and duplicated segments of the ITD are below. The proximal breakpoint at the second base of the stop codon (TGA) alters it to a TTA (leucine). (**d**) Schematic of predicted BCOR protein sequences from ITD-positive CCSKs demonstrating the clustering of all ITDs within the C-terminal PUFD domain. ITD types I–V were numbered based on genomic breakpoints and ITD sequence (Table 1). The BCOR wild-type protein sequence (amino acids (aa) 1,701–1,755) is shown on top with the predicted protein sequence of each ITD-positive case below. Parental segments that have been duplicated are shown in green and the ITDs in red. Novel junctional amino acids (bold black font, underlined) were introduced by the ITDs in cases 385T and 504T. A stretch of 14 residues (aa 1,724–1,737) is common to every ITD type. ANK, ankyrin repeats; BBD, BCL6-binding domain.

On closer inspection, WES tumour sequencing reads harbouring the variant *BCOR* allele were found to have adjacent soft clipping (Supplementary Fig. 1a). Notably, analysis of the aligned RNA-seq reads from all three tumours revealed similar soft-clipped subsequences (Supplementary Fig. 1b). Since soft clipping by mapping algorithms may be indicative of reads spanning genomic breakpoints of structural variations^12^, we analysed the clipped *BCOR* sequences using the Basic Local Alignment Search Tool^13^ and discovered in-frame ITDs within exon 15 of *BCOR* in all three cases (Table 1). Local realignment of discordant mate pairs showed a distinct focal increase in read coverage corresponding to the ITDs (Fig. 1a and Supplementary Fig. 2), which were subsequently confirmed by targeted PCR and sequencing (Fig. 1b,c).

Targeted DNA sequencing of *BCOR* exon 15 in a validation cohort of 11 additional CCSKs (Supplementary Table 1) revealed in-frame ITDs in 8 additional tumours (Table 1 and Fig. 1b,d), resulting in an overall mutation frequency of 11/14 (78%), including tumours from 7 of 9 males and 4 of 5 females. Sequencing of cloned ITD alleles identified 5 distinct ITD types with overlapping genomic breakpoints within exon 15 of *BCOR*, ranging in size from 87 to 114 bp (Table 1). The ITDs are predicted to involve amino acids 1,701–1,755 within the C-terminal PUFD (polycomb-group RING finger homologue (PCGF) Ub-like fold discriminator)^14^ domain of the protein (Fig. 1d). In one case, the ITD in *BCOR* was interrupted by a 3-bp insertion (Table 1), as has been observed for ITDs in the *FLT3* tyrosine kinase^15^.

All *BCOR* ITDs were confirmed to be absent from patient-matched peripheral blood and/or adjacent normal kidney samples, when available (Fig. 1b and Supplementary Table 1). Testing of two metastatic relapsed lesions revealed identical ITDs as in the primary tumour (Fig. 1b). In males, who are hemizygous at the *BCOR* locus, the wild-type allele was virtually undetectable (Fig. 1b), suggesting that ITD acquisition is an early event in CCSK tumorigenesis. *BCOR* ITDs were not found in a cohort of other childhood renal tumours (18 Wilms tumours and 9 congenital mesoblastic nephromas) and soft-tissue sarcomas (*n*=10). Analysis of a non-overlapping cohort of 13 CCSKs subjected to transcriptome sequencing as part of the National Cancer Institute's Therapeutically Applicable Research to Generate Effective Treatments (TARGET) initiative (http://ocg.cancer.gov/programs/target) identified ITDs within exon 15 of *BCOR* in 12 of 13 cases (Supplementary Data 3), including one additional ITD type (type VI), which were verified by realigning the RNA-seq reads to ITD-specific modified reference transcriptomes (Methods and Supplementary Fig. 3) and by local realignment of discordant mate-pair mapping to the *BCOR* transcript (Supplementary Fig. 2). In total, therefore, *BCOR* ITDs were identified in 23/27 (85%) of CCSKs analysed.

### Expression of *BCOR* mRNA and protein in tumours

**Figure 2:**
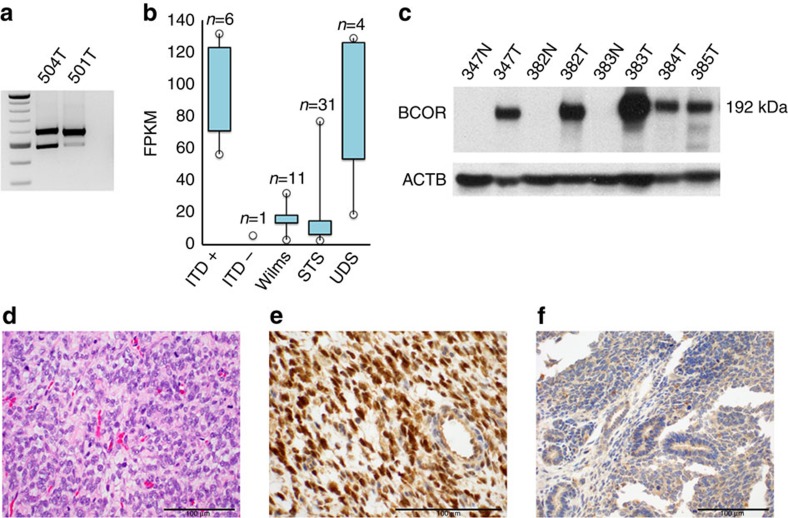
*BCOR* expression in CCSKs. (**a**) Targeted RT–PCR of a segment of the *BCOR* transcript (exons 14 and 15) in CCSKs from female patients demonstrated expression of both the wild-type product (491 bp) and a larger product corresponding to the mutant (ITD) allele, confirming expression of the ITD from the active X chromosome. (**b**) Box-and-whisker plot of estimated *BCOR* transcript abundance from RNA-seq data in ITD-positive CCSKs (ITD+) demonstrating high expression of *BCOR* in comparison to an ITD-negative CCSK (ITD−), Wilms tumours (Wilms) and assorted soft-tissue sarcomas (STS). UDS with *BCOR*–*CCNB3* fusions also had upregulated *BCOR* expression. Bar representing 25th–75th percentile, line representing the maximum and minimum values. (**c**) Immunoblot using an antibody to full-length BCOR protein^10^ demonstrated a 192-kDa product corresponding to the predicted size of BCOR^10^ in ITD-positive CCSK tumours (T) but not in matched normal kidney samples (N). ACTB, beta-actin. (**d**) Haematoxylin-and-eosin-stained section of CCSK showing classic histologic pattern. Immunohistochemistry with BCOR antibody demonstrated strong nuclear staining in the tumour cells in (**e**) CCSKs but not in (**f**) Wilms tumours.

Targeted reverse transcription–PCR (RT–PCR) of an intron-spanning segment of the *BCOR* transcript (exons 14 and 15; Supplementary Table 3) confirmed expression of the mutant allele in all ITD-positive tumours tested (*n*=4), including two from female patients, suggesting that the mutant allele had not been silenced through X-inactivation (Fig. 2a). Transcript abundance estimation of RNA-seq data from 6 ITD-positive CCSKs using Cufflinks^16^ showed strong upregulation of *BCOR* as compared with 11 Wilms tumours, 31 assorted sarcomas and 1 ITD-negative CCSK (Fig. 2b). *BCOR* transcripts were similarly expressed at high levels in the TARGET consortium CCSKs. RSEM (RNA-Seq by Expectation-Maximization)^17^ was used to estimate the relative fractions of mutant and wild-type *BCOR* transcripts in ITD-positive tumours by remapping unaligned RNA-seq reads to individual tumour-specific synthetic reference transcriptomes, revealing that 96–100% of *BCOR* expression was contributed by mutant transcripts (data not shown). Notably, the four undifferentiated sarcomas (UDS) harbouring *BCOR*–*CCNB3* fusions^18^ tested were also shown to have high *BCOR* expression (Fig. 2b).

These findings were corroborated on the protein level by immunoblotting BCOR in five ITD-positive cases and three adjacent normal kidney specimens. An antibody to the full-length protein confirmed upregulated BCOR expression in the ITD-positive CCSKs compared with normal kidney (Fig. 2c). Similarly, immunohistochemistry showed strong and diffuse nuclear staining in all CCSKs tested (*n*=6) but not in Wilms tumours (Fig. 2d–f) or congenital mesoblastic nephromas (data not shown).

### Transcriptome analysis of CCSKs

**Figure 3:**
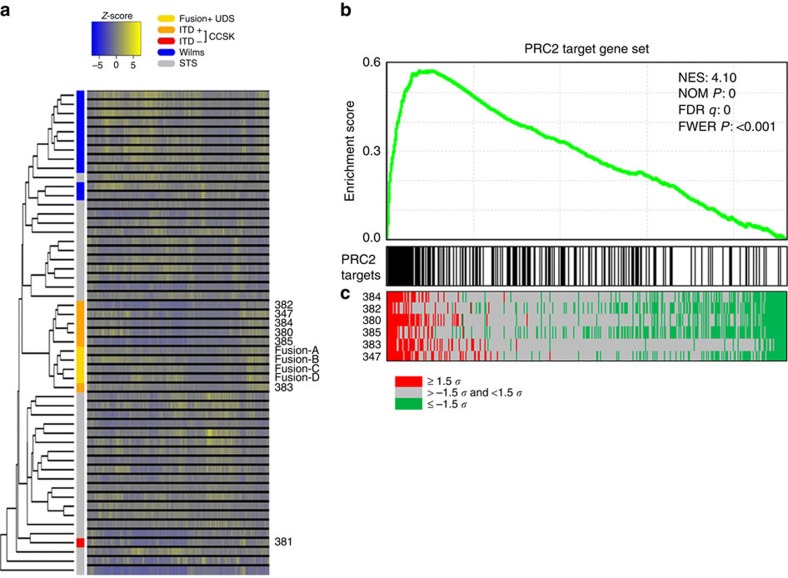
Transcriptome profiling of renal tumours and soft-tissue sarcomas. (**a**) Unsupervised hierarchical clustering revealed ITD-positive CCSKs (*n*=6) to cluster separately from Wilms tumours (*n*=11), the lone ITD-negative CCSK (case 381) and other soft-tissue sarcomas (STS; *n*=31). The transcriptomes of *BCOR*–*CCNB3* fusion-positive UDS (*n*=4) cluster together and are closely related to those of the ITD-positive CCSKs. The heatmap reflects the variance in gene expression relative to the mean across all samples (*n*=53) with upregulated genes in yellow and downregulated genes in blue. (**b**) GSEA of CCSKs revealed significant upregulation of PRC2 targets. Enrichment score computed by the GSEA algorithm (y axis) is plotted against the PRC2 target genes (x axis) rank ordered by the degree of differential expression in ITD+ CCSKs compared with Wilms tumours. High-ranking PRC2 target genes are upregulated in CCSKs relative to Wilms tumours (**c**) Heatmap showing expression values of PRC2 targets in individual ITD-positive CCSK cases as a deviation from the mean in Wilms tumours in units of s.d. The PRC2 target gene set (BENPORATH_PRC2_TARGETS) includes genes with promoter regions bound to H3K27me3, SUZ12 and EED in human H9 ES cells. *p*, nominal *p*-value; FDR, false discovery rate; FWER, family-wise error rate.

Unsupervised hierarchical clustering using RNA-seq data revealed similarities between the transcriptomes of ITD-positive CCSKs and *BCOR*–*CCNB3* fusion-positive UDS and suggested that RNA-expression programs in these tumours are distinct from those of Wilms tumours, other sarcomas or the single ITD-negative CCSK tested (Fig. 3a). When the CCSKs from both our study cohort and the TARGET consortium cohort were analysed separately by unsupervised clustering, the ITD-negative CCSKs appeared to be distinct from ITD-positive tumours in both studies (Supplementary Fig. 4). Genes that were found to be differentially expressed between ITD-positive CCSKs and Wilms tumours (Supplementary Data 4) were utilized for gene-set enrichment analysis (GSEA) using MSigDB-curated gene sets^19^. The 10 most significantly enriched gene sets identified were related to PRC2 targets or associated with the trimethylated histone H3 on Lys27 (H3K27me3) mark (Fig. 3b,c and Supplementary Table 4) (FWER *P* value <0.001). Significant enrichment of the Hedgehog signalling pathway (FWER *P* value <0.001) and downstream targets *CCND1* and *PDGFRA* was also found in CCSKs (Supplementary Figs 5 and 6), confirming previous reports^20^.

All of the *BCOR* ITDs identified in this study target the C-terminal PUFD domain through which BCOR physically interacts with PCGF1 (ref. 14) within a variant PCR1 (PRC1.1/BCOR complex)^21^,^22^. This complex also includes KDM2B and the E3-ubiquitin ligase RNF2 that directly monoubiquitylates lysine-119 of histone H2A (H2AK119ub1; refs 23, 24). Given the observed enrichment of PRC2 targets from our GSEA analysis and the fact that PRC1-dependent H2AK119ub1 is a recruitment mark for PRC2 at distinct genomic loci^25^,^26^, we investigated the PRC2 target set in more detail. Although PRC2 target genes were both upregulated (*n*=125) and downregulated (*n*=70; *P*<0.05; *t*-test, one-tailed, unequal variance) in CCSKs (Fig. 3c and Supplementary Data 5) relative to Wilms tumours, analysis using GSEA and the Database for Annotation, Visualization and Integrated Discovery (DAVID)^27^,^28^ toolset also revealed a highly significant enrichment for homeobox proteins (FWER *P*<0.001; Supplementary Fig. 7) and upregulation of distinct classes of homeobox proteins, which are the canonical targets of polycomb-mediated repression.

## Discussion

In conclusion, the discovery of highly recurrent *BCOR* ITDs in CCSK highlights the power of unbiased next-generation sequencing (NGS) to identify genetic drivers of tumorigenesis. ITDs are an unusual class of genetic alterations, previously reported as oncogenic gain-of-function mutations, most notably in the receptor tyrosine kinases *FLT3* and *KIT*^15^,^29^,^30^. As demonstrated in the current study, this rarity may be due in part to the use of computational mapping algorithms that discard discordant mate pairs in NGS data. Systematic application of comprehensive ITD-detection algorithms to tumour genome data will be necessary to more rigorously evaluate the prevalence of this class of genetic alterations. The discovery of *BCOR* ITDs in the vast majority of CCSKs, but not in Wilms tumours, offers the potential for a molecular diagnostic test for these cancers.

Recently, Ueno-Yokohata *et al*.^31^ published a study describing the identification of recurrent *BCOR* ITDs in CCSKs using conventional RT–PCR to investigate the correlation between *BCOR* promoter CpG hypomethylation and the observed high expression of *BCOR* in CCSKs. The observed frequency and types of *BCOR* ITDs is very similar to the findings reported in this article. In both studies, CCSK tumours harbouring *BCOR* ITDs exhibit high expression of *BCOR* mutant transcripts and protein. Understanding the mechanism by which *BCOR* expression is upregulated in these tumours will require further studies, including assessing the activity of the PRC1.1/BCOR complex in the context of mutant BCOR. Although BCOR co-purifies with KDM2B^22^, it is unknown if that involves direct interaction with KDM2B or other members of the PRC1.1 complex; regardless, it is intriguing to speculate that the ITDs disrupt the structure and/or function of the complex leading to derepression at target promoters, including that of *BCOR* itself, which is a target bound by KDM2B in mouse embryonal cells^32^.

Consistent with possible disruption of the PRC1.1/BCOR complex in CCSKs, transcriptome profiling revealed widespread upregulation of PRC2 targets in these tumours, suggesting disruption of polycomb regulation (Fig. 3c) as a potential pathogenic mechanism in CCSK. Expression profiling revealed ITD-positive CCSKs to share significant similarity with *BCOR*–*CCNB3* fusion-positive UDS, a soft tissue and bone tumour type not previously considered related to CCSK but now shown to have in common somatic alterations affecting the C-terminal PUFD domain of BCOR. The fact that both CCSKs and *BCOR*–*CCNB3* fusion-positive sarcomas are defined by mutations in an X-linked gene may underlie the known male predominance of these two tumour types. Further studies are necessary to understand the effects of the *BCOR* ITD in cell and animal models and uncover the underlying consequences of this genetic alteration on PRC function and the mechanism of CCSK oncogenesis.

## Methods

### Patient enrolment and study design

Informed consent was obtained for enrolment of patient 347 on the NHGRI and NCI-funded Clinical Sequencing Exploratory Research programme BASIC3 study at Baylor College of Medicine (BCM) and Texas Children's Hospital (TCH) under a BCM Institutional Review Board-approved protocol. Tumour specimens from 13 additional CCSK patients were identified in the TCH Pathology database (*n*=7) and at the University of Texas Southwestern (UT-SW) Medical Center (*n*=6) and analysed under BCM and UT-SW Institutional Review Board-approved protocols. Informed consent was obtained for all prospectively enroled patients. Study protocols allowed waiver of consent for use of de-identified specimens obtained from archived cases. Basic clinical features of all cases (*n*=14) are described in Supplementary Table 1. In two CCSK patients, biopsies of relapsed metastatic tumours were also available and included in the genomic analysis. All CCSK cases enroled in the study were reviewed independently by four pathologists (D.R., K.W.E., M.J.H. and A.R.). Wilms tumour (*n*=18), congenital mesoblastic nephroma (*n*=9) and miscellaneous other childhood bone and soft-tissue sarcomas (*n*=31) were included as controls and tested under approved protocols as described above.

### DNA and RNA isolation

Genomic DNA from fresh-frozen tissue (for WES libraries and PCR) or formalin-fixed paraffin-embedded (FFPE) tissue (for targeted BCOR sequencing) was isolated using the QiaAmp DNA Mini kit (Qiagen) according to the manufacturer's protocol. Total RNA was extracted from fresh-frozen tissue (for RNA-seq libraries and RT–PCR) using the mirVana miRNA isolation kit (Life Technologies). Total RNA from FFPE tissue (for targeted BCOR sequencing) was extracted using the RecoverAll Total Nucleic Acid Isolation kit (Life Technologies).

### Library preparation for exome and transcriptome sequencing

WES was performed for patient 347 on tumour (T) and matched normal (N) genomic DNA from peripheral blood. Exome libraries for the T/N pair were generated and sequenced on a single lane of an Illumina HiSeq 2000 as previously described^33^, yielding a mean coverage of × 186 (N) and × 203 (T) and a target base coverage of 97.5% at × 20. Whole-transcriptome RNA sequencing (RNA-seq) for seven CCSK samples (347T, 380T, 381T, 382T, 383T, 384T and 385T) was performed using 1 μg total RNA to prepare strand-specific poly-A+ RNA-seq libraries for sequencing on the Illumina platform. Library preparation details except with the following modifications are the same as described previously^18^. Purified mRNA from total RNA was fragmented by heat at 94 °C for 3–4 min. Libraries were prepared as described previously^18^ and pooled in equimolar amounts (2 libraries per pool) and sequenced on a HiSeq 2000 or 2500 to generate reads in paired-end mode (2 × 100-bp reads). On average, for these seven samples, 83.7 million paired reads (155–201 million total reads) were generated per sample. Summary sequencing statistics for the RNA-seq data are presented in Supplementary Data 1.

### Somatic mutation analysis from WES data

Data analysis including alignment, variant calling and annotation was performed using the semi-automated Mercury data analysis pipeline to generate annotated vcf files as previously described^33^. After merging the tumour vcf and the corresponding germline vcf files, the following filters were applied for calling somatic (tumour-specific) variants: variant ratio >0.05, total tumour coverage >50, variant coverage in tumour >6 and variant coverage in normal <4. Somatic variants were additionally annotated with information from the COSMIC database (Catalog of Somatic Mutations In Cancer; v59) to include frequency of variant position hits in COSMIC, nearby hits and most common tumour types affected. Whole-exome and transcriptome sequence data of tumours for which patients (or their parent/legal guardian) gave consent for the deposit of their information have been deposited in dbGaP under the accession code phs001026.

### RNA-seq analysis

RNA-seq reads were aligned using STAR v2.3.0e (ref. 34) to an index of hg19 that included GENCODE v16 gene annotation (http://www.gencodegenes.org/archive_stats.html). Alignment files were processed using Picard tools v1.54 (http://picard.sourceforge.net/), and the final BAM files indexed using SAMtools index v0.1.11 (ref. 35). RNA-seq run quality was assessed using the RNA-SeQC package^36^ using the same GENCODE 16.gtf file and hg19 reference as was used to create the index. Transcript quantification was performed using Cufflinks^16^ v2.02 running in quantification mode against the GENCODE v16.gtf file. FPKM (Fragments Per Kilobase of Exon Per Million Fragments Mapped) values were used for relative abundance estimation.

To estimate the ratio of *BCOR* ITD to wild-type *BCOR* from RNA-seq reads, the RefSeq RNA transcriptome (dated 4 June 2014) for protein-coding sequences was modified by replacing all *BCOR* transcripts with two competing *BCOR* sequences for alignment: one wild-type sequence containing only the last six exons of *BCOR* and the first 304 bp of the 3′-untranslated region (UTR), and another six exon transcript with the corresponding ITD bases added and the same 304 bp of 3′-UTR. All other RefSeq transcripts remained unmodified. The RNA-seq reads were aligned and quantified to this modified transcriptome with RSEM^17^ (version 1.2.17) and Bowtie2 (ref. 37).

Detection of fusion genes was performed using deFuse^38^ as previously published^18^. Briefly, high-quality FASTQ files were subjected to analysis with default options and filters (Supplementary Data 2). Predicted read-through candidates and nominated candidates, which did not retain an open reading frame were discarded, and the remaining nominated candidates were ranked based on location of fusion breakpoints.

### Discordant mate-pair mapping

FASTQ files were aligned to the RefSeq RNA transcriptome using Bowtie2. Discordant mate pairs, where one mate mapped to *BCOR* and the other was unmapped, were extracted and a pileup plot was produced with Integrative Genomics Viewer (IGV). Unmapped mates were remapped to the genome using BLAT to reveal the junction regions of the *BCOR* ITD.

### BCOR ITD analysis from TARGET project transcriptome data

FASTQ files from the TARGET project CCSK cases (Supplementary Data 3) were first aligned to the RefSeq RNA transcriptome using Bowtie2. The FASTQ files of samples showing evidence of *BCOR* ITDs (insertions in exon 15 of *BCOR*) were remapped to a modified transcriptome with both *BCOR* ITD and wild-type *BCOR* transcripts using RSEM as described above. Reads spanning the ITDs are displayed in Supplementary Fig. 3. Discordant read mapping, as described above, was also applied to verify ITD peaks (Supplementary Fig. 2).

### Hierarchical clustering

Unsupervised hierarchical clustering was implemented in R (ref. 39) using the hclust procedure with average (Unweighted Pair Group Method with Arithmetic Mean) agglomeration and (1—Spearman correlation) distance. The dendrogram and heatmap were graphed with heatmap.2 (gplots package: http://cran.r-project.org/web/packages/gplots/index.html). For the clustering of 6 ITD-positive CCSK cases, 1 ITD-negative CCSK, 4 *BCOR*–*CCNB3* fusion-positive sarcomas, 11 Wilms tumours and 31 other sarcomas, mRNA expression FPKM values for 12,775 genes were included after filtering for HGNC protein-coding genes with average expression above 1.5 FPKM and coefficient of variation (CV) above 0.3 over the set of all 53 tumours.

The same criteria were used for the clustering of gene expression in TARGET cohort CCSKs (http://target.nci.nih.gov/dataMatrix/). Briefly, mRNA expression FPKM values for 3,355 genes were included after filtering for HGNC protein-coding genes with average expression above 1.5 FPKM and CV above 0.3 over the set of all 13 tumours.

### Gene-set enrichment analysis

GSEA calculations were performed with the GSEA programme (v. 2.0.14) (ref. 19). The Broad Molecular Signatures Database (MSigDB v5.0) set c2 (curated gene sets) was used. For each tumour type comparison, mRNA expression FPKM values were included after filtering for HGNC protein-coding genes with average expression above 1.5 FPKM and CV above 0.3 over the set of all tumours in the comparison. In addition, ribosomal protein L family (RPL) and ribosomal protein S family (RPS) ribosomal genes were removed. For ITD-positive CCSKs (6 cases) compared with Wilms tumours (11 cases), the GSEA analysis involved 4,538 gene sets and the expression of 11,513 genes. The GSEA programme was run with 10,000 randomized gene sets for statistical significance estimation, and the default signal-to-noise metric between the two phenotypes was used to rank all genes.

In addition, GSEA comparisons were run using the same parameters with the MSigDB c2 collections of gene sets supplemented with a set of 170 genes corresponding to the ‘homeobox' protein family downloaded from the UniProtKB human proteome (UniProt proteome: UP000005640) using the following parameters: ‘family and domains'—‘protein family'—homeobox; ‘organism'—human; and ‘reviewed' status as ‘yes'. All *P* values reported by GSEA as zero represent values lower than 10^−4^ (1/10,000 permutations).

### Targeted PCR and sequencing for ITD detection

Targeted PCR was performed on cases and controls with specific primers (BCOR-ITD_F/BCOR-ITD_R and BCOR-ITD_Intron 14_F/BCOR-ITD_3UTR_R, Supplementary Table 3) designed to detect the ITDs, followed by agarose gel electrophoresis. To validate and map the ITDs, the ITD fragments were either directly sequenced or cloned using the TOPO-TA cloning kit (Life Technologies) followed by sequencing using BigDye Terminator v3.1 chemistry on a 3730xl DNA Analyzer (Life Technologies).

### RT–PCR to examine expression of the *BCOR* ITD

Total RNA was isolated from available fresh-frozen tumours of two female patients (501T and 504T). Total RNA (500 ng) was used for RT–PCR with the SuperScript III First-Strand Synthesis System (Life Technologies) and specific intron-spanning primers (Supplementary Table 3) were designed on exons 14 (forward) and 15 (reverse) to target the 3′ coding sequence of the mature *BCOR* mRNA (NM_001123385.1). Expression of ITD in the tumours of males was also validated using the same primers.

### Immunoblot assays

Total protein was extracted from fresh-frozen tissue from seven CCSK tumours and three adjacent normal kidney tissue using RIPA buffer in the presence of cOmplete, Mini, EDTA-free protease inhibitor (Roche). Protein electrophoresis and immunoblotting were performed with 30 μg of total protein on NuPAGE 3–8% Tris-Acetate gels (Life Technologies) and transferred onto a polyvinylidene difluoride membrane following standard protocols. BCOR and β-actin were detected using a mouse anti-BCOR antibody (Abcam, ab88112, 1:1,000 dilution) and a rabbit polyclonal anti-β-actin antibody (Abcam, ab8227, 1:10,000).

### Immunohistochemistry

Immunohistochemistry was performed on 5-μm-thick FFPE sections using the automated Leica Bond system. Epitope retrieval was carried out using Novocastra Bond Epitope Retrieval Solution 1, pH 6.0 (Leica, AR9961). Sections were then incubated for 15 min with two different BCOR antibodies (mouse anti-BCOR antibody, Abcam ab88112, or rabbit polyclonal anti-BCOR, BCOR-184 from V. Bardwell), both at 1:500 dilutions, that produced similar results. Results depicted (Fig. 2e,f) are using the mouse antibody. The detection kit used was the Bond Polymer Refine Detection (Leica, DS9800), incubation with post primary for 8 min, polymer for 8 min, DAB for 10 min and haematoxylin for 5 min.

## Additional information

**Accession codes:** Whole-exome and transcriptome sequence data of tumours have been deposited in dbGaP under the accession code phs001026. The TARGET project CCSK transcriptome data have been deposited in dbGAP under the accession code phs000466.

**How to cite this article:** Roy, A. *et al*. Recurrent internal tandem duplications of *BCOR* in clear cell sarcoma of the kidney. *Nat. Commun.* 6:8891 doi: 10.1038/ncomms9891 (2015).

## Supplementary Material

Supplementary Information Supplementary Figures 1-7 and Supplementary Tables 1-4

Supplementary Data 1 RNA-seq QC metrics/coverage data

Supplementary Data 2 List of fusion genes identified in CCSK cases by deFuse and filters applied

Supplementary Data 3 BCOR internal tandem duplications in CCSK tumors identified in this study

Supplementary Data 4 Genes/transcripts differentially expressed in ITD+CCSK and Wilms tumors

Supplementary Data 5 Expression of PRC2 targets enriched in ITD+ CCSK relative to Wilms tumors

## Figures and Tables

**Table 1:** *BCOR* internal tandem duplications identified in CCSK patients.

**Sample**	**Patient age (years)**	**Patient sex**	**ITD length (bp)**	**ITD genotype**	Nucleotide (gDNA)*	**Nucleotide (cDNA)**	**Amino acid (protein)**
347T	0.9	M	96	Type I	chrX:39,911,364–39,911,459	c.5171_5266dup	p.L1724_W1755dup
383T	1.1	M	96	Type I	chrX:39,911,364–39,911,459	c.5171_5266dup	p.L1724_W1755dup
385T	1.9	M	87	Type IV	chrX:39,911,406–39,911,492†	c.5138_5224dup	p.V1741_E1742insV+p.L1713_V1741dup
380T	1.2	M	93	Type II	chrX:39,911,374–39,911,466	c.5164_5256dup	p.E1722_D1752dup
382T	1.8	M	90	Type III	chrX:39,911,405–39,911,494	c.5136_5225dup	p.D1712_V1741dup
384T	2.2	M	96	Type I	chrX:39,911,364–39,911,459	c.5171_5266dup	p.L1724_W1755dup
474T	0.6	M	93	Type II	chrX:39,911,374–39,911,466	c.5164_5256dup	p.E1722_D1752dup
499T	3.5	F	96	Type I	chrX:39,911,364–39,911,459	c.5171_5266dup	p.L1724_W1755dup
501T	1.6	F	93	Type II	chrX:39,911,374–39,911,466	c.5164_5256dup	p.E1722_D1752dup
504T	2.7	F	114	Type V	chrX:39,911,418–39,911,531	c.5099_5212dup	p.G1738E+p.V1701_L1737dup
624T	2.9	F	96	Type I	chrX:39,911,364–39,911,459	c.5171_5266dup	p.L1724_W1755dup

cDNA, complementary DNA; gDNA, genomic DNA.

^*^Genomic coordinates as per NCBI build 37/hg19, negative strand.

^†^The ITD in case 385T also included a 3-bp junctional insertion (c.5224_5225insTGT).
