# Supplementary material for: Recurrent internal tandem duplications of BCOR in clear cell sarcoma of the kidney
Source: Nat Commun. 2015 Nov 17;6:8891. doi: 10.1038/ncomms9891 (PMC4660214; doi:10.1038/ncomms9891)
Supplement: Supplementary Information — Supplementary Figures 1-7 and Supplementary Tables 1-4 [file ncomms9891-s1.pdf]

Supplementary Figure 1a

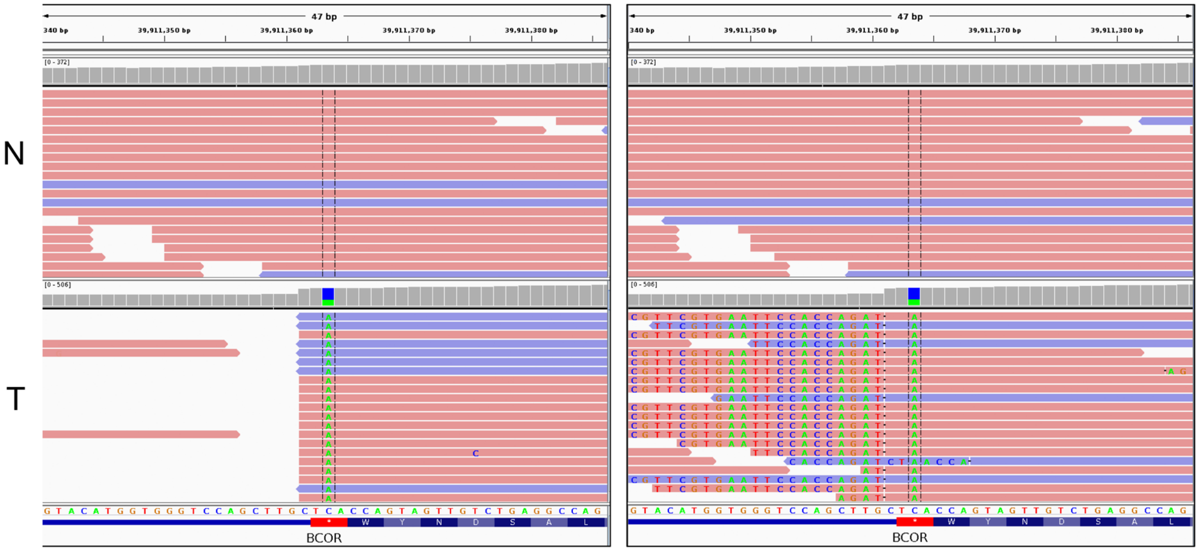

Supplementary Figure 1b

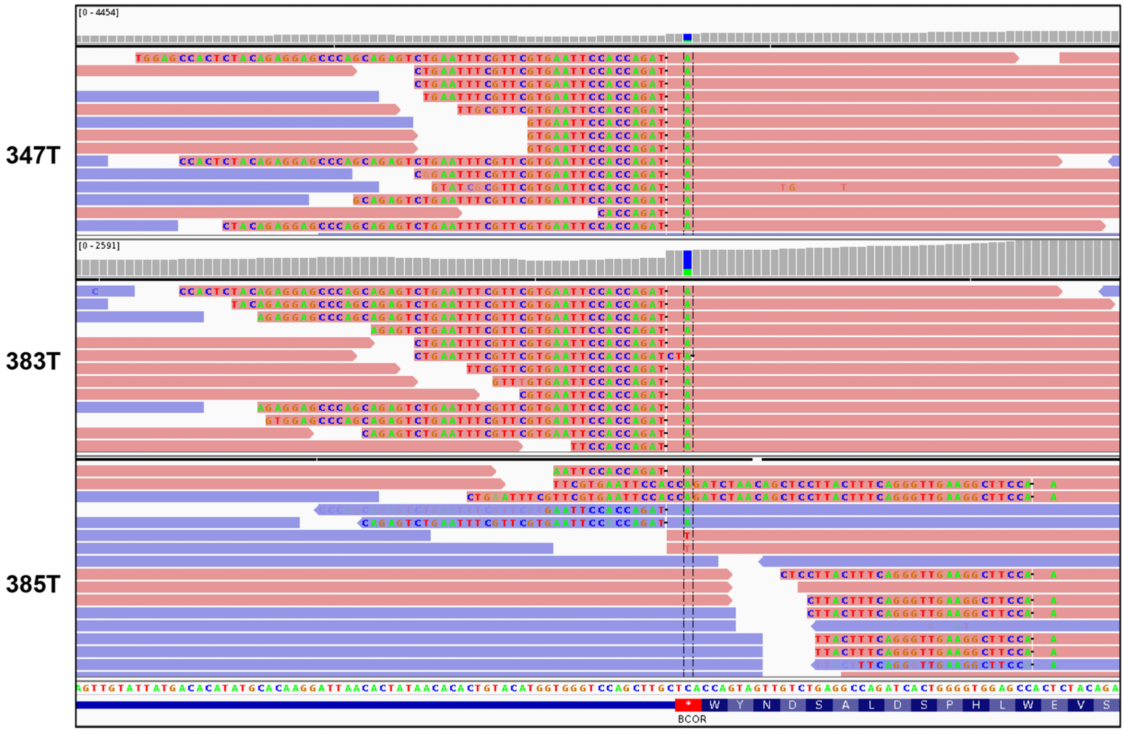

Supplementary Figure 1a. Whole-exome sequencing of paired tumor (T) and matched germline sample (N) from 347T shows the putative stop-loss variant detected in *BCOR* only within the tumor reads and not in the germline (left panel), with adjacent soft-clipping masking subsequences (right panel) that correspond to the ITD. **Supplementary Figure 1b.** Soft-clipped subsequences in *BCOR* exon 15 RNA-seq reads in each of three discovery CCSK cases. In 347T (and in 383T), the proximal breakpoint is at the termination codon as detected by whole-exome sequencing of 347T (see Supplementary Fig. 1a).

Supplementary Figure 2.

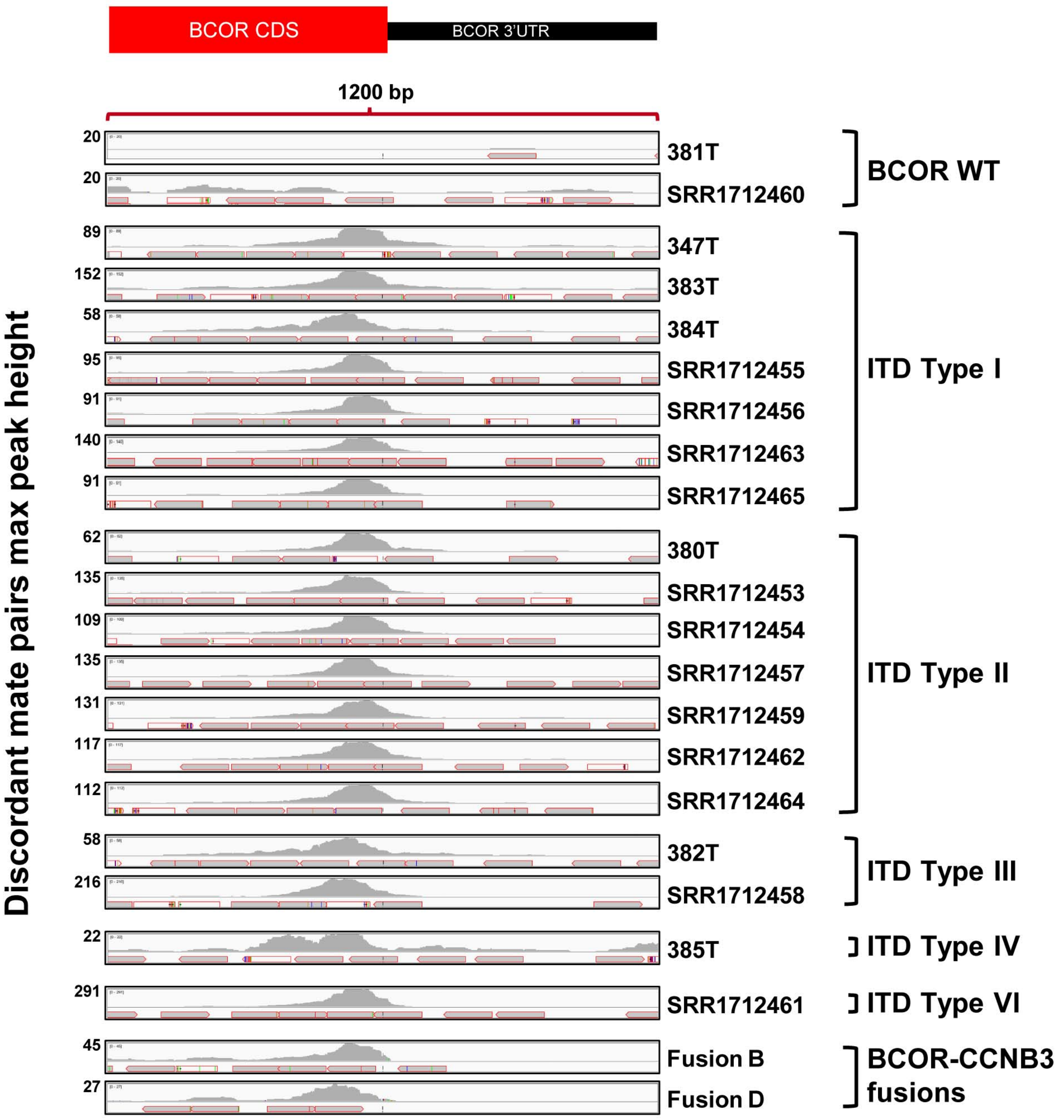

Supplementary Figure 2. Discordant mate-pair mapping based on RNA-seq. A pile-up plot of discordant mate-pairs, where one mate mapped to *BCOR* and the other was unmapped, was produced with IGV (Integrative Genomics Viewer). The plots show distinct peaks corresponding to ITD segments in ITD+ cases but not in CCSKs with wild-type *BCOR* (BCOR WT at the top). Pile-up plots of the two *BCOR-CCNB3* fusion sarcomas analyzed showed peaks that were similar to those of ITD+ cases; these reflect mate-pair reads where one read mapped to *BCOR* mRNA and the other to *CCNB3* mRNA. No RNA-seq data was available for ITD Type V, which was genotyped by Sanger sequencing.

Supplementary Figure 3a

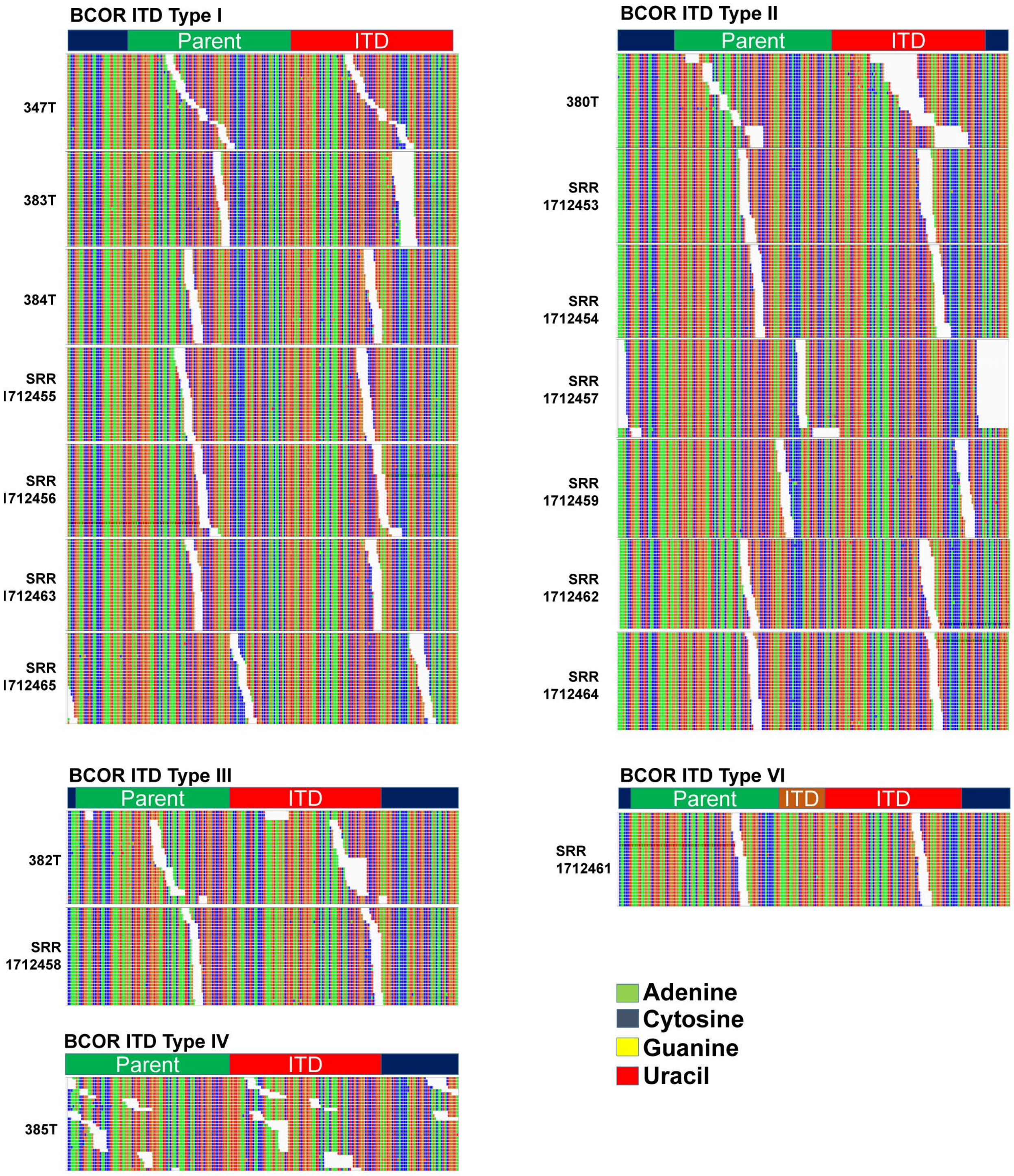

Supplementary Figure 3a. RSEM was used to align RNA-seq reads to modified reference transcriptomes, including predicted *BCOR* ITDs in addition to all RefSeq transcripts (see methods and text). Reads spanning the proximal breakpoint at the junction of the parental (green) and the ITD (red) segments match perfectly to ITD type-specific-modified transcriptomes. When mapped to published RefSeq transcripts only, these reads undergo either soft-clipping (see Supplementary Fig. 1) or are discarded as discordant mates, which can be detected by mapping unpaired discordant mate-pairs (see Supplementary Fig. 2). *BCOR* ITDs in the TARGET project CCSK cases (numbered with the 'SRR' prefix) were inferred bioinformatically. In SRR1712461, a short stretch of an ITD (brown) is juxtaposed in between the parental (green) and the distal ITD (red) segments. No RNA-seq data was available for ITD type V, which was genotyped by Sanger sequencing.

Supplementary Figure 3b

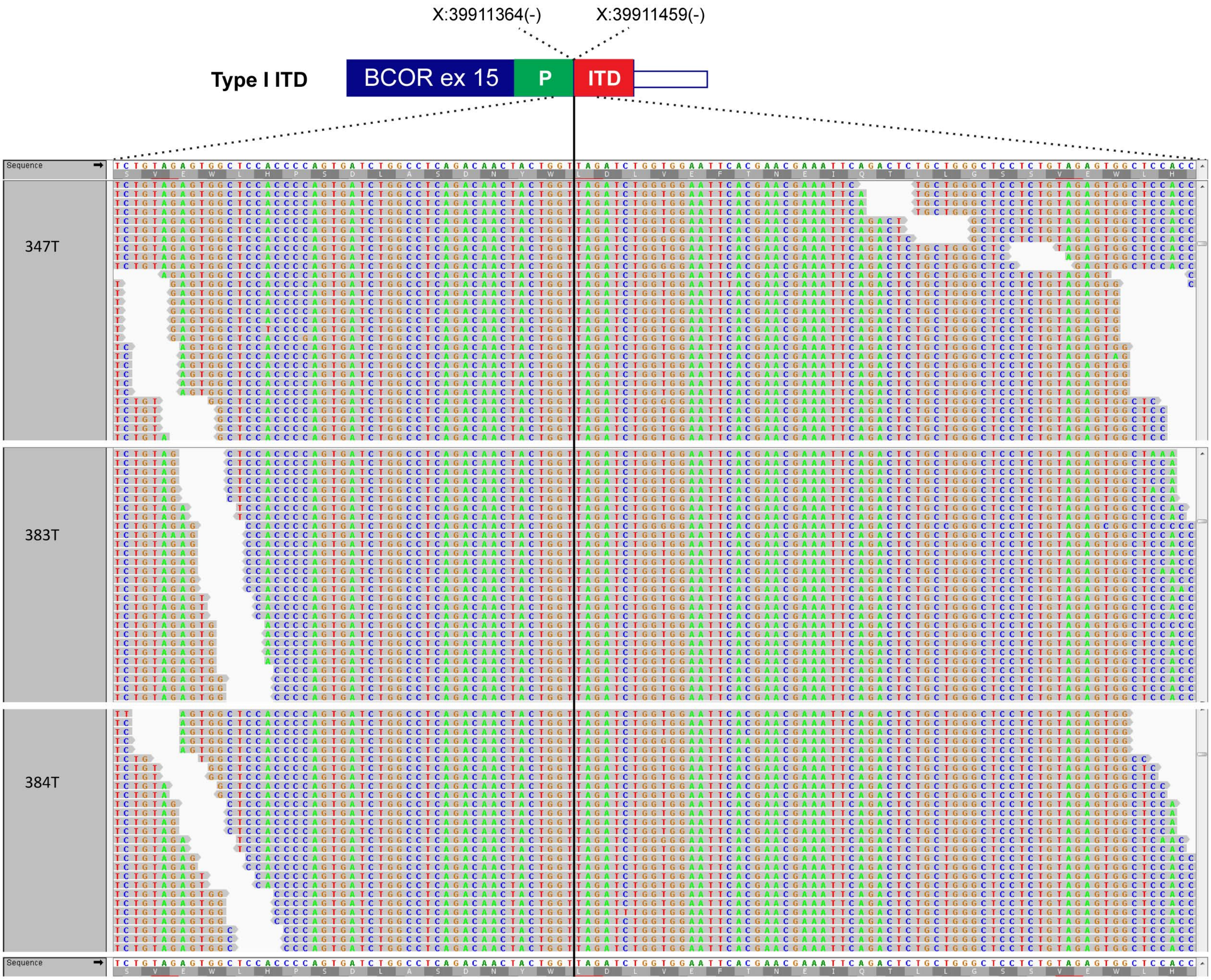

Supplementary Figure 3b. Sequence context surrounding the proximal breakpoint of type I ITDs in the validation cohort. P, parental segment; ITD, internal tandem duplication. hg19 genomic coordinates (minus strand).

Supplementary Figure 3c

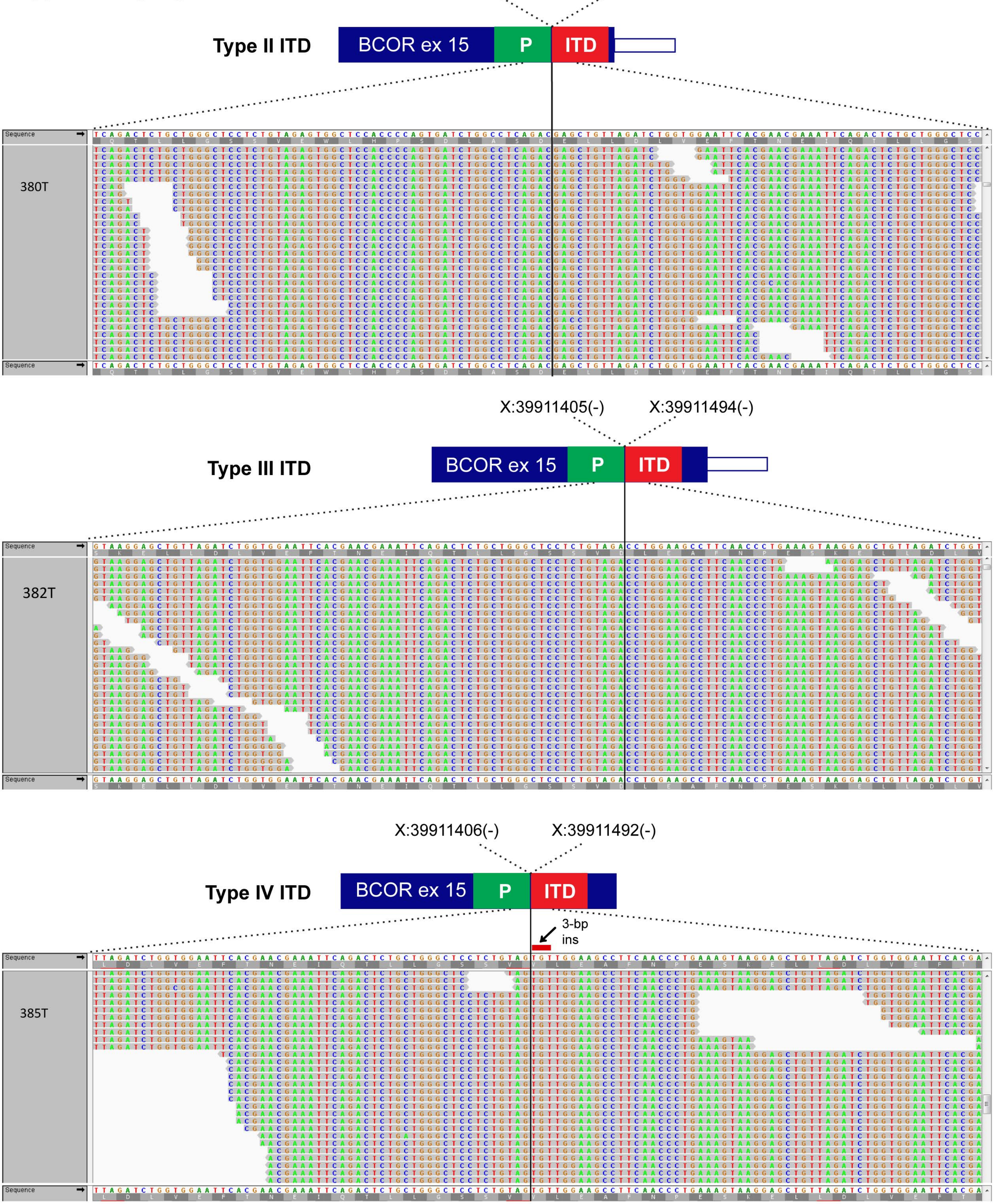

Supplementary Figure 3c. Sequence context surrounding the proximal breakpoints of type II, III and IV ITDs in the validation cohort. A 3-bp insertion interrupts the type IV ITD. P, parental segment; ITD, internal tandem duplication. hg19 genomic coordinates (minus strand).

Supplementary Figure 3d

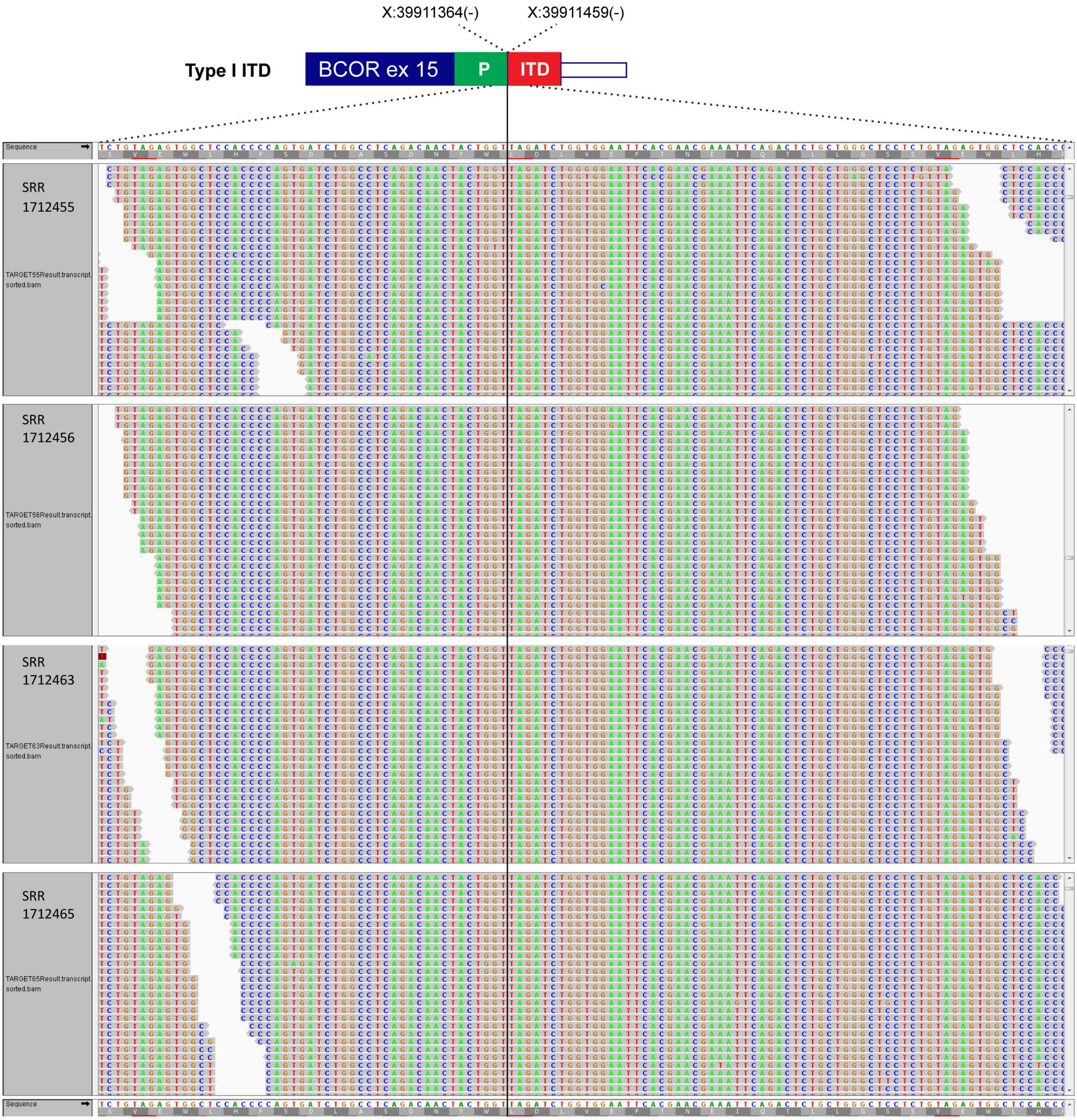

Supplementary Figure 3d. Sequence context surrounding the proximal breakpoint of type I ITDs in the TARGET consortium cohort. Parental segment; ITD, internal tandem duplication. hg19 genomic coordinates (minus strand).

Supplementary Figure 3e

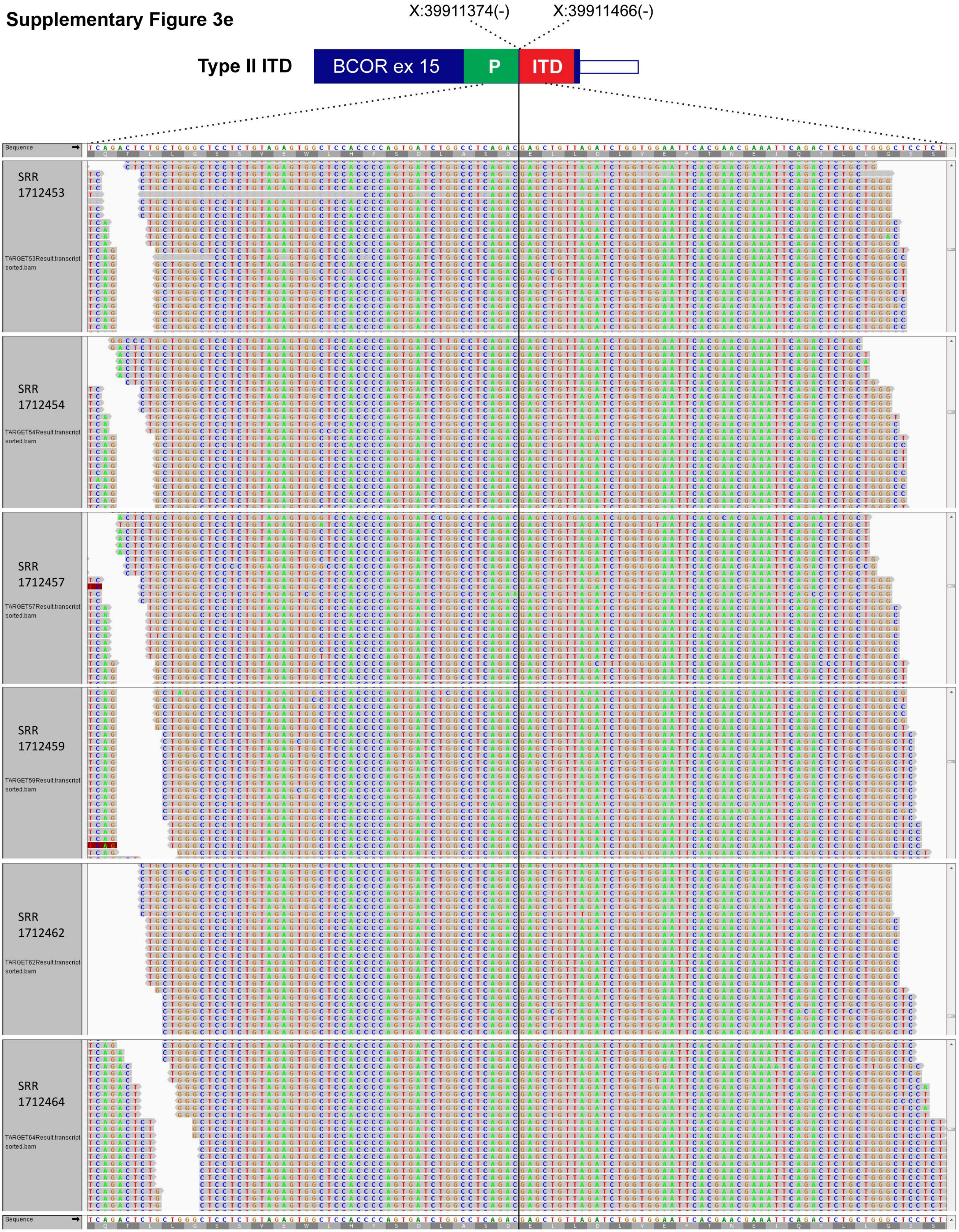

Supplementary Figure 3e. Type II ITDs in the TARGET consortium cohort.

Supplementary Figure 3f

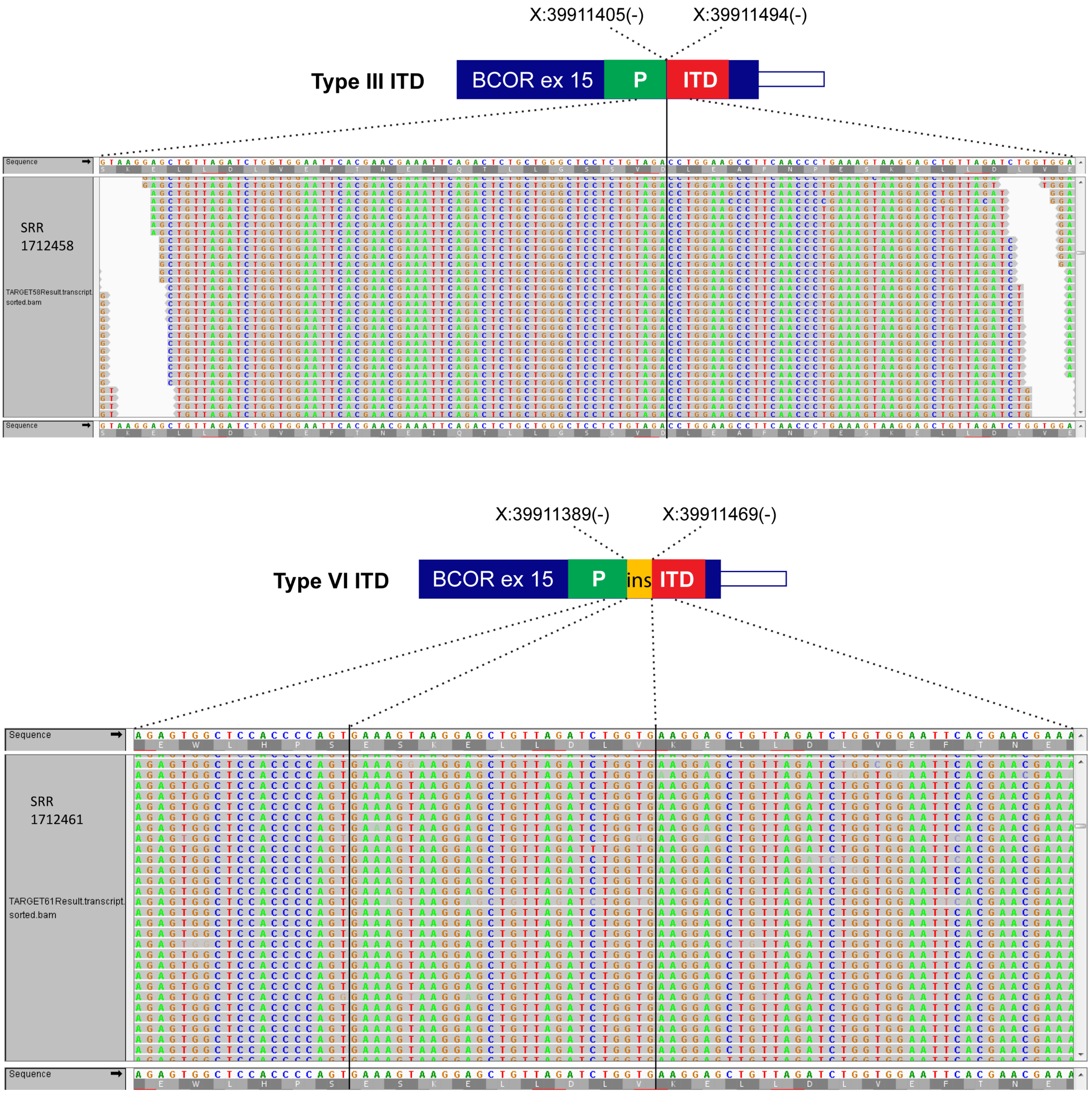

Supplementary Figure 3f. Sequence context surrounding the proximal breakpoints of type III and VI ITDs in the TARGET consortium cohort. A 27-bp insertion interrupts the type VI ITD. P, parental segment; ITD, internal tandem duplication; ins, insertion. hg19 genomic coordinates (minus strand).

Supplementary Figure 4

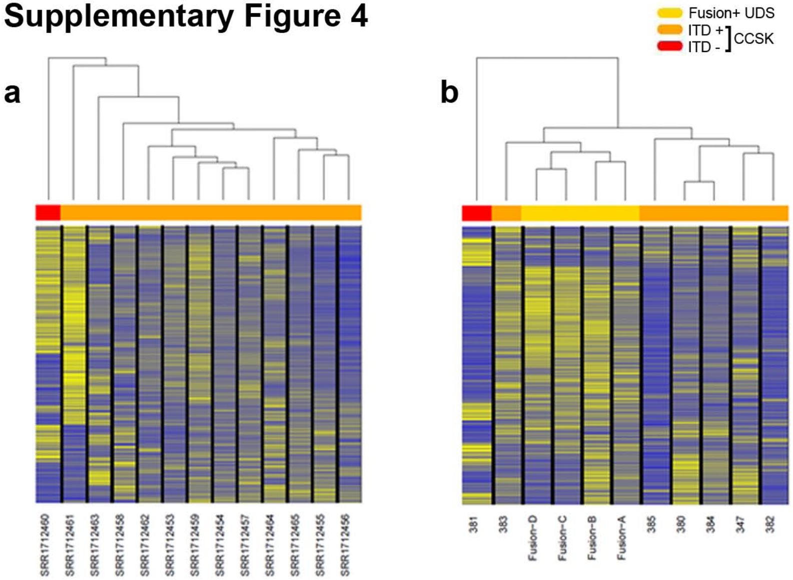

Supplementary Figure 4. Unsupervised hierarchical clustering revealed ITD-positive CCSKs from both the TARGET cohort (a) and the local cohort (b) to cluster separately from the ITD-negative CCSKs.

Supplementary Figure 5

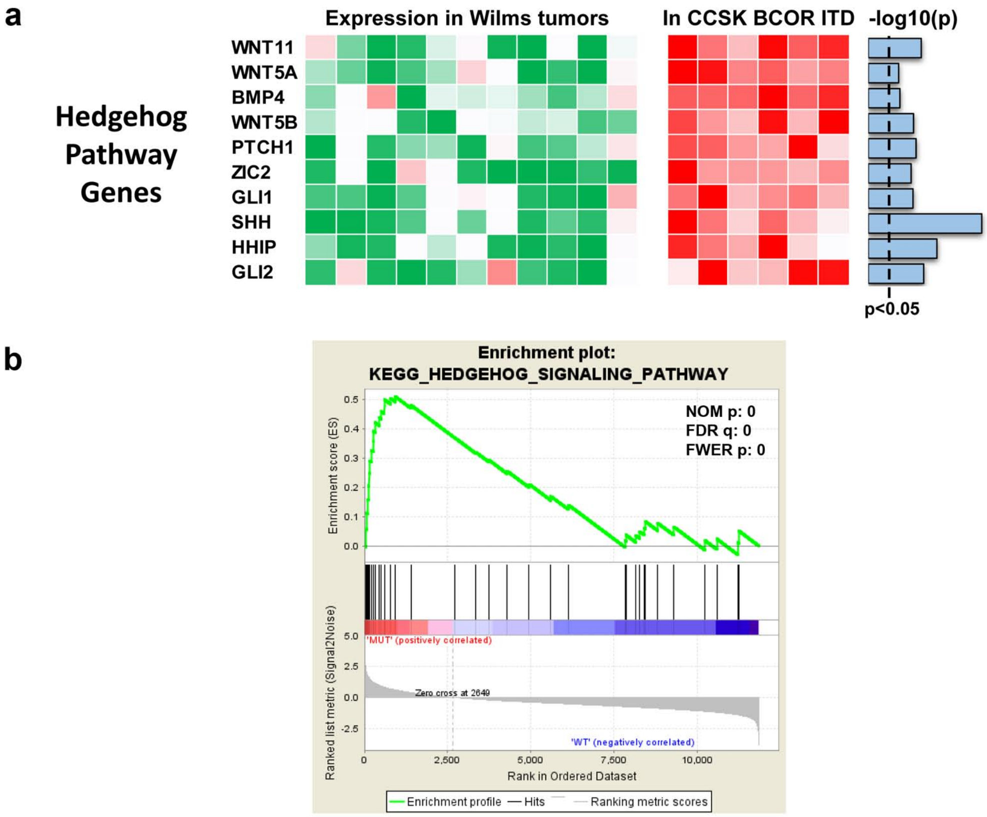

Supplementary Figure 5. Activation of Sonic Hedgehog (Shh) pathway in CCSK. (a) Heatmap depicting high expression (red) of Shh signaling pathway transcripts in 6 ITD+ CCSK tumors relative to 11 Wilms tumors. (b) GSEA analysis demonstrating significant enrichment for Shh pathway members in ITD+ CCSK relative to Wilms tumors. The Y-axis in the upper panel shows the enrichment score as computed by GSEA. The X-axis demonstrates genes (black lines) rank-ordered by the degree of differential expression in ITD+ CCSK (‘MUT’) relative to Wilms tumors (‘WT’), with the highest ranking genes being upregulated in CCSKs. The bottom panel shows the intensity of differential expression by a ranked signal/noise metric.NOM p, Nominal p-value; FDR, False Discovery Rate; FWER, Family-wise Error rate.

Supplementary Figure 6

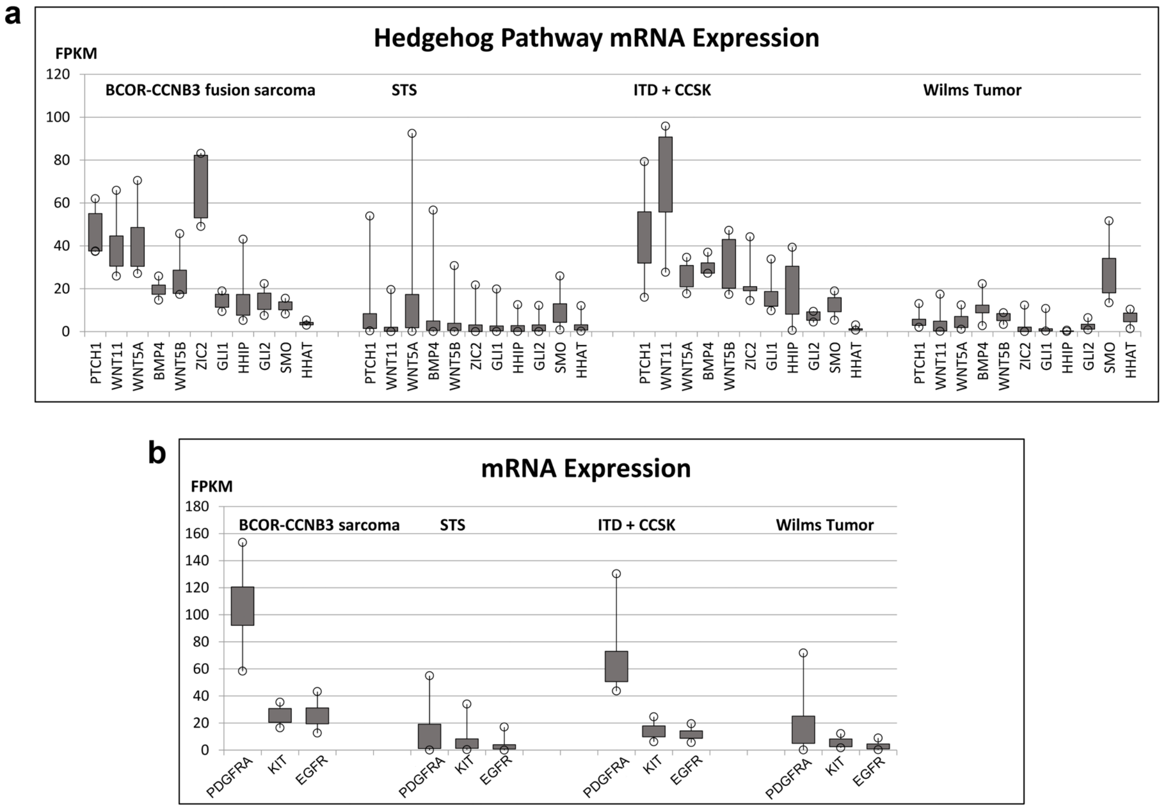

Supplementary Figure 6. Expression profiling of Hedgehog pathway members by RNA-seq. (a) ITD+ CCSK and *BCOR-CCNB3* fusion-positive sarcomas both show similar high expression of SHH pathway members. (b) The SHH pathway target, *PDGFRA*, is also upregulated in both ITD+ CCSKs and the fusion-positive sarcomas. ITD+CCSK (n=6), BCOR-CCNB3 sarcomas (n=4), STS (n=31), Wilms tumors (n=11). Boxes represent the 25th to 75th percentile values. Open circles mark the lowest and highest values.

Supplementary Figure 7

a

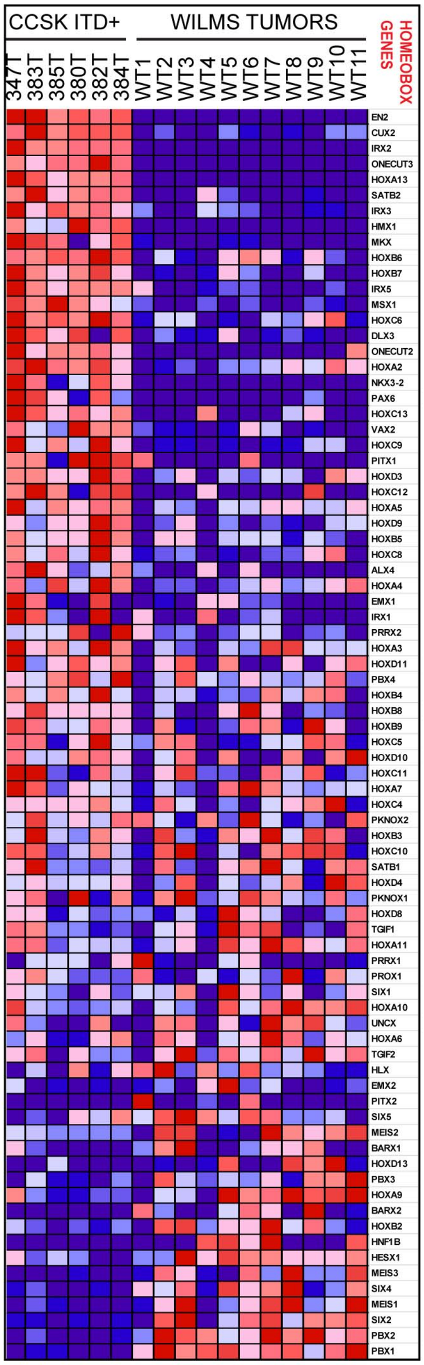

b

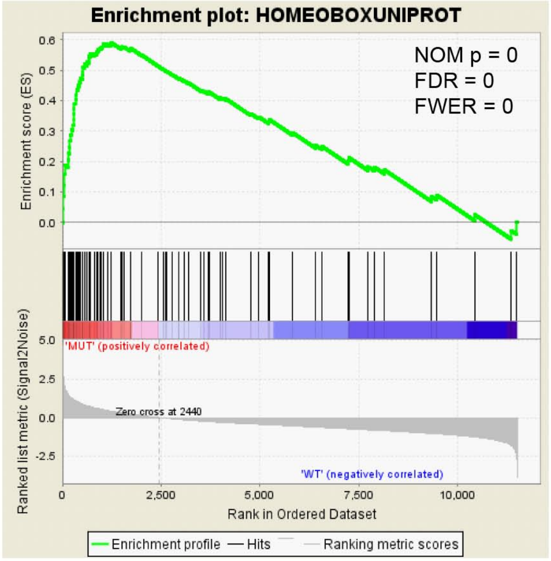

Supplementary Figure 7. (a) Heatmap of expression levels of a curated set of homeobox genes differentially expressed in ITD+ CCSKs relative to Wilms tumors (red: overexpression , blue: under expression). (b) GSEA analysis revealed highly significant enrichment for homeobox genes in ITD+ CCSKs (NES, Normalized enrichment score = 3.38). The Y-axis in the upper panel shows the enrichment score as computed by GSEA. The X-axis demonstrates genes (black lines) rank-ordered by the degree of differential expression in ITD+ CCSK ('MUT') relative to Wilms tumors ('WT'), with the highest ranking genes being upregulated in CCSKs. The bottom panel shows the intensity of differential expression by a ranked signal/noise metric. NOM p, Nominal p-value; FDR, False Discovery Rate; FWER, Family-wise Error rate.

Supplementary Table 1. Clinical data and molecular testing performed on CCSK cases

	Case	Age (yrs)	Sex	Samples available		Testing performed		*BCOR* status
				Tumor*	Normal	RNA-seq	WES	
Discovery cohort	347	0.9	M	Y	B, K	Y	Y	ITD
	383	1.1	M	Y	K	Y	N	ITD
	385	1.9	M	Y	K	Y	N	ITD
Validation cohort	380	1.2	M	Y	N	Y	N	ITD
	381	2.3	F	Y	N	Y	N	wt
	382	1.8	M	Y	K	Y	N	ITD
	384	2.2	M	Y	N	Y	N	ITD
	474	0.6	M	Y*	N	N	N	ITD
	495	1.9	M	Y	K	N	N	wt
	497	1.1	M	Y	N	N	N	wt
	499	3.5	F	Y	K	N	N	ITD
	501	1.6	F	Y*	K	N	N	ITD
	504	2.7	F	Y	K	N	N	ITD
	624	2.9	M	Y	K	N	N	ITD

Age at time of original diagnosis. Targeted denotes targeted BCOR testing performed. *Tumor samples also available from metastatic relapse. WES, whole exome sequencing; RNA-seq, RNA-sequencing; Y, yes; N, no; B, blood; K, kidney; M, male; F; female; ITD, internal tandem duplication; wt, wild-type.

Supplementary Table 2. Somatic mutations identified by WES in case 347T

Gene	Chr	Position (hg19)	REF	ALT	RefSeq Transcript	Exon	DNA	Amino acid	Domain	Variant Class	Variant Fraction (%)
BCOR	X	39911363	C	A	NM_001123385	15	c.5171_5266dup	p.L1724_W1755dup	PCGF Ub-like fold Discriminator	Duplication (non-frameshift)	33.91
AZGP1	7	99565862	G	A	NM_001185	3	c.C529T	p.R177W	Class I Histocompatibility antigen, domains alpha 1 and 2	Missense	6.03
USP7	16	8995093	T	C	NM_003470	20	c.2048-2A>G		ICP0-binding domain	Splicing	47.45

Ref, Reference allele; Alt, alternate allele

Supplementary Table 3. Primers used for PCR and RT-PCR analysis of BCOR

Primer Name	Primer sequence	Expected product size (bp)
BCOR-ITD_F	5'-TCTTGTTCTCTTGCTCCAAAGAC -3'	252*
BCOR-ITD_R	5'-TGTAATAGTGTCCTTTCTTTACAGA -3'	
BCOR-ITD_Intron 14_F	5'-TGGTCCACTTGGGTTGGTAG -3'	441
BCOR-ITD_3UTR_R	5'-TGACACATATGCACAAGGATTAACA -3'	
BCOR-RT_F	5'-GGCTATGATGTTTTAGCCAACC -3'	491
BCOR-RT_R	5'-TGACACATATGCACAAGGATTAACA -3'	

*288 bp with M13 tags as used in this study.

Supplementary Table 4. Gene sets most significantly enriched by GSEA analysis in ITD+ CCSK relative to Wilms tumors

GSEA GENE SET NAME	GENE SET SIZE	ES	NES	NOM p-value	FDR q-value	FWER p-value
BENPORATH_PRC2_TARGETS	312	0.57	3.87	0	0	0
MIKKELSEN_NPC_HCP_WITH_H3K27ME3	151	0.56	3.67	0	0	0
MEISSNER_NPC_HCP_WITH_H3K4ME2_AND_H3K27ME3	174	0.56	3.57	0	0	0
MIKKELSEN_IPS_WITH_HCP_H3K27ME3	41	0.69	3.40	0	0	0
HOMEOBOXUNIPROT	80	0.59	3.38	0	0	0
MIKKELSEN_MCV6_HCP_WITH_H3K27ME3	200	0.50	3.35	0	0	0
MIKKELSEN_NPC_HCP_WITH_H3K4ME3_AND_H3K27ME3	129	0.50	3.10	0	0	0
MIKKELSEN_MEF_HCP_WITH_H3K27ME3	201	0.46	3.05	0	0	0
KEGG_BASAL_CELL_CARCINOMA	35	0.62	2.93	0	0	0
MEISSNER_BRAIN_HCP_WITH_H3K27ME3	113	0.47	2.88	0	0	0
RODRIGUES_NTN1_TARGETS_UP	14	0.78	2.84	0	0	0
MEISSNER_NPC_HCP_WITH_H3K4ME3_AND_H3K27ME3	95	0.49	2.84	0	0	0
PEREZ_TP53_AND_TP63_TARGETS	159	0.39	2.61	0	4.39E-04	0.0033
MEISSNER_NPC_HCP_WITH_H3K27ME3	33	0.56	2.61	0	4.45E-04	0.0035

GSEA, Gene Set Enrichment Analysis; ES, Enrichment Score; NES, Normalized Enrichment Score; NOM p-value, Nominal p-value; FDR, False Discovery Rate; FWER, Familywise-Error Rate
